# Steering Does Affect Biophysical Responses in Asynchronous, but Not Synchronous Submaximal Handcycle Ergometry in Able-Bodied Men

**DOI:** 10.3389/fspor.2021.741258

**Published:** 2021-10-25

**Authors:** Cassandra Kraaijenbrink, Riemer J. K. Vegter, Nils Ostertag, Luc Janssens, Yves Vanlandewijck, Lucas H. V. van der Woude, Heiko Wagner

**Affiliations:** ^1^Department of Movement Science, Institute for Sport and Exercise Sciences, University of Münster, Münster, Germany; ^2^Department of Human Movement Sciences, University Medical Centre Groningen, University of Groningen, Groningen, Netherlands; ^3^Peter Harrison Centre for Disability Sport, School of Sport, Exercise and Health, Loughborough University, Loughborough, United Kingdom; ^4^Electrical Engineering (ESAT) TC, Campus Group T Leuven, KULeuven, Leuven, Belgium; ^5^Department of Rehabilitation Sciences, Faculty of Movement and Rehabilitation Sciences, KULeuven, Leuven, Belgium; ^6^Department of Physiology, Nutrition and Biomechanics, The Swedish School of Sport and Health Sciences (GIH), Stockholm, Sweden; ^7^Department of Rehabilitation Medicine, University Medical Centre Groningen, University of Groningen, Groningen, Netherlands

**Keywords:** cyclic exercise, upper body exercise, ergometry, crank mode, mechanical efficiency, handcycle technique

## Abstract

Real-life daily handcycling requires combined propulsion and steering to control the front wheel. Today, the handcycle cranks are mostly mounted synchronously unlike the early handcycle generations. Alternatively, arm cycle ergometers do not require steering and the cranks are mostly positioned asynchronously. The current study aims to evaluate the effects of combining propulsion and steering requirements on synchronous and asynchronous submaximal handcycle ergometry. We hypothesize that asynchronous handcycling with steering results in the mechanically least efficient condition, due to compensation for unwanted rotations that are not seen in synchronous handcycling, regardless of steering. Sixteen able-bodied male novices volunteered in this lab-based experiment. The set-up consisted of a handcycle ergometer with 3D force sensors at each crank that also allows “natural” steering. Four submaximal steady-state (60 rpm, ~35 W) exercise conditions were presented in a counterbalanced order: synchronous with a fixed steering axis, synchronous with steering, asynchronous with a fixed axis and asynchronous with steering. All participants practiced 3 × 4 mins with 30 mins rest in between every condition. Finally, they did handcycle for 4 mins in each of the four conditions, interspaced with 10 mins rest, while metabolic outcomes, kinetics and kinematics of the ergometer were recorded. The additional steering component did not influence velocity, torque and power production during synchronous handcycling and therefore resulted in an equal metabolically efficient handcycling configuration compared to the fixed condition. Contrarily, asynchronous handcycling with steering requirements showed a reduced mechanical efficiency, as velocity around the steering axis increased and torque and power production were less effective. Based on the torque production around the crank and steering axes, neuromuscular compensation strategies seem necessary to prevent steering movements in the asynchronous mode. To practice or test real-life daily synchronous handcycling, a synchronous crank set-up of the ergometer is advised, as exercise performance in terms of mechanical efficiency, metabolic strain, and torque production is independent of steering requirements in that mode. Asynchronous handcycling or arm ergometry demands a different handcycle technique in terms of torque production and results in higher metabolic responses than synchronous handcycling, making it unsuitable for testing.

## Introduction

Handcycling is a popular form of outdoor propulsion for persons who are dependent on their upper body for locomotion during and after rehabilitation (Kraaijenbrink et al., 2020b). To practice or test handcycle performance, the arm crank ergometer is often used as an indoor alternative, especially in early rehabilitation (Krops et al., 2017; Kouwijzer et al., 2018b; Bresnahan et al., 2019; Williams et al., 2019; Brizuela et al., 2020). Outdoor handcycling requires steering, hence the front wheel and fork must rotate freely around the steering axis. Today's handcycles are equipped with a synchronous (parallel) crank setting, which is different from the early handcycles with asynchronous crank sets (cranks 180° out of phase) (Müller and Müller, 1949; Fink, 1976; Cummins and Gladden, 1983; van der Woude et al., 1986) or in today's arm crank ergometers. Arm crank ergometry (ACE) is performed without steering, as the crank system is often fixed to the wall or floor in a stable position and is indeed often seen with the cranks in an asynchronous mode. Research in both handcycling and arm crank ergometry have shown that the response to both crank modes can be quite different (Mossberg et al., 1999; van der Woude et al., 2000, 2008; Abel et al., 2003; Dallmeijer et al., 2004; Goosey-Tolfrey and Sindall, 2007; Bafghi et al., 2008; Smith et al., 2008; Faupin et al., 2011; Kraaijenbrink et al., 2020a). The additional constraint of steering is assumed to be crucial (van der Woude et al., 2000, 2008; Dallmeijer et al., 2004; Bafghi et al., 2008; Kraaijenbrink et al., 2020a), but has not been investigated to date.

During submaximal treadmill-based handcycling, which apart from air resistance is quite similar to straight over ground handcycling, a clear preference for the synchronous mode exists in terms of metabolic response, which is shown in various studies with able-bodied inexperienced men (van der Woude et al., 2000, 2008; Dallmeijer et al., 2004; Bafghi et al., 2008; Kraaijenbrink et al., 2020a). By solely changing the crank mode from synchronous to asynchronous at an equal belt speed and equal submaximal external power output level, oxygen uptake (+19%), ventilation (+17%), breathing frequency (+4%), and local perceived discomfort (+49%) increase. Subsequently, the mechanical efficiency is lower (relatively −13%) for asynchronous handcycling (van der Woude et al., 2000, 2008; Dallmeijer et al., 2004; Bafghi et al., 2008; Kraaijenbrink et al., 2020a). In asynchronous handcycling, the asymmetric upper body movements require more arm and trunk stabilization effort to combine accurate steering with propulsion compared to synchronous handcycling (Dallmeijer et al., 2004). After a practice period, we previously have found a different handcycle technique between the two modes. In the synchronous mode, participants show to have a pulling strategy, i.e., they pull when the crank is farthest away and show no upward push. In the asynchronous mode, participant also pull the crank when it is farthest away, but they also exert a negative propulsion force and a more radial directed force when the crank is directed toward the chest. Hence, they hold the handlebar in a stable position rather than pushing the crank upwards in order to control for unwanted steering movements caused by the propulsion force exerted at the other crank (Kraaijenbrink et al., 2020a). Asynchronous handcycling consequently cost more energy and is less efficient than synchronous handcycling in this group of able-bodied novices.

For arm crank exercise it is unclear whether the synchronous or asynchronous crank mode is preferential to reduce the energy costs. For able-bodied males, Hopman et al. (1995) have found a relative increase of 10% in gross mechanical efficiency when switching from synchronous to asynchronous ACE at 30 W. But at 60 and 90 W, no significant differences between both modes was determined (Hopman et al., 1995). Also Mossberg et al. (1999) have not identified any significant differences between crank modes across multiple submaximal intensities (up to 100 W), for able-bodied men as well as men and a woman with paraplegia. Goosey-Tolfrey and Sindall (2007) have encountered a preference toward asynchronous ACE at either 60 or 80 W, in an experienced population of trained male wheelchair dependent athletes [with disabilities ranging from amputation to (in)complete spinal cord injury (L4-5 up to C2-5)]. A decrease in oxygen uptake (−9%) and therefore an increase in the gross mechanical (+12%) and net efficiency (+16%) have been seen when shifting to asynchronous ACE (Goosey-Tolfrey and Sindall, 2007). These three studies have all been conducted with an arm ergometer that has been fixed to the wall or floor. When specifically trained male and female handcyclists perform in their own handcycle and the front wheel is fixed in the ergometer (Cyclus II), like in the study of Abel et al. (2003), a preference toward a synchronous crank mode seems to exist, suggesting training effects for this mode. At 30, 60 and 90 W, an increase in oxygen uptake (+11%) has been found when converting from synchronous to asynchronous ACE, which indicates a decrease in mechanical efficiency (Abel et al., 2003). In contrast to handcycling, there is no need for active control of rotation around the steering axis for regular arm crank exercise. One has to view the results of arm ergometry studies in this context. All in all, at a low power output, a small preference toward an asynchronous crank mode seems to be expected for novices in arm crank ergometry.

Based on the previous experimental literature, the necessity of steering while propelling the crank set in handcycling, complicates the latter and seems to affect the metabolic cost, especially in the asynchronous mode (van der Woude et al., 2000, 2008; Dallmeijer et al., 2004; Bafghi et al., 2008; Kraaijenbrink et al., 2020a). How simultaneous steering and propulsion during submaximal handcycling affects the metabolic cost, kinetics, and kinematics of steady state propulsion has not been addressed yet. The current study involves a combined physiological and biomechanical comparison of simulated handcycling in a specially designed handcycle ergometer that allows for natural steering characteristics with two degrees of freedom (2DoF), being rotation around the crank axis and rotation around the steer axis (Verellen et al., 2012a). The steer axis can be fixed (1 DoF condition) or released (2 DoF condition) prior to experimenting. Four submaximal steady-state exercise conditions have been presented: synchronous with a fixed steering axis, synchronous with steering requirements, asynchronous with a fixed steering axis and asynchronous with steering requirements. Subsequently, the aim of the current study is to investigate the effects of steering requirements on mechanical efficiency, the velocity, as well as the torque and power production patterns and their differences between both crank modes during submaximal simulated handcycling in a group of able-bodied male novices. To ensure an equal level of experience in all conditions, a series of practice sessions has been provided in each of the four conditions for all participants. Based on the treadmill studies, in which asynchronous handcycling is less mechanically efficient and power production less effective than synchronous handcycling, we have hypothesized that an asynchronous crank mode with steering will create the least efficient condition during submaximal steady state propulsion. However, as soon as the steering axis is fixed, asynchronous handcycling is expected to be more efficient, as the need for stabilization is canceled out. In line with the literature on arm crank exercise, we expect asynchronous handcycling without steering to be as efficient, if not more efficient than synchronous handcycling.

## Materials and Methods

### Participants

Sixteen able-bodied male novices (age: 25 ± 5 years, mass: 78.30 ± 10.90 kg, height: 1.80 ± 0.07 m, arm length: 0.61 ± 0.04 m) volunteered in the study, after signing an informed consent subsequent to being informed about the study procedure and aims. Inclusion criteria were male, age of 18 years or older, non-smoker, no shoulder injuries in the last 3 months, and no experience in handcycling or arm crank exercise. All participants were healthy and could take part in exercise as was made sure of with the physical activity readiness questionnaire (PAR-Q; Thomas et al., 1992). The participants refrained from alcohol 24 h and from caffeine in the morning prior to the experiment. This study was approved by the local ethics committee of the Faculty of Psychology and Sport Science at the University of Muenster (2019-15-CK-EA).

### Experimental Protocol

**Figure 1:**
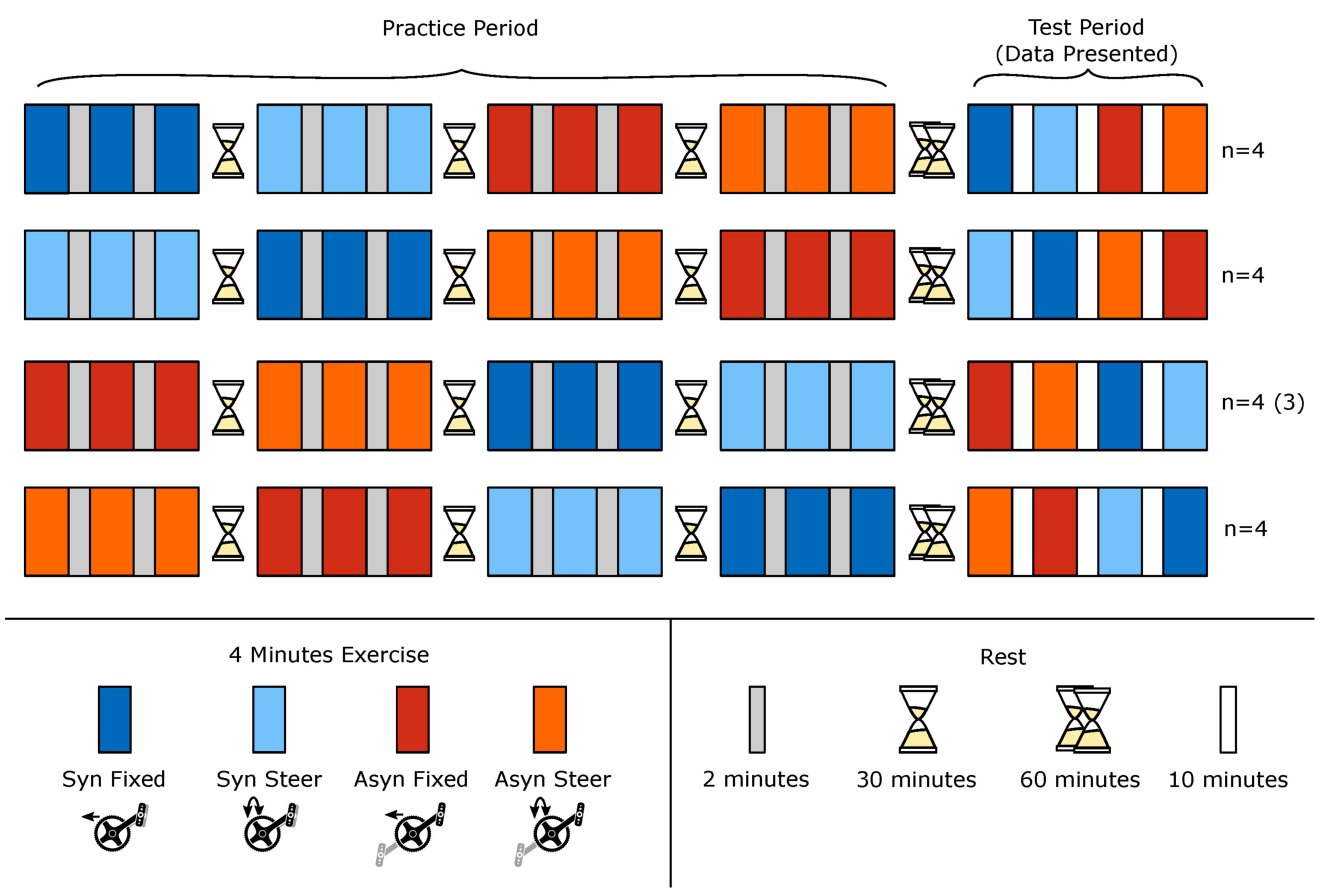
Schematic overview of the experimental protocol. The experimental protocol consisted of a practice and test period; the latter is reported in this study. Participants practiced 3 × 4 mins in every condition with minimal 2 mins rest in between. In the test period, the participants performed every condition once for 4 mins with 10 mins rest in between. The external power output level was set at the same value of 35 W for all conditions. The first 45 s of the last minute of each of the four modes in the test period were used for analysis, to ensure a physiological steady state.

The experimental protocol consisted of a practice and a test period and was conducted on one single day for each participant (Figure 1). The following four exercise conditions were presented to all participants in a counterbalanced order: (1) cranks mounted synchronously with a fixed steering axis (Syn Fixed); (2) cranks mounted synchronously with free rotation around steering axis (Syn Steer); (3) cranks mounted asynchronously with a fixed steering axis (Asyn Fixed); and (4) cranks mounted asynchronously with free rotation around the steering axis (Asyn Steer). To familiarize themselves with the experimental set-up and to learn to handcycle in each condition, the participants practiced every condition for a duration of 12 mins divided in 3 blocks of 4 mins with minimal 2 mins rest in between. Within the first 12 mins of exercise the propulsion technique can be adapted as previously shown in several forms of wheeled mobility (Vegter et al., 2014; Kraaijenbrink et al., 2020a). In the practice period, the participants had a minimum of 30 mins rest before starting the next condition. In these 30 mins, the participants could drink water. Between the practice and the test period, the participants had a minimum of 1-h rest. In this rest period the participant ate a light snack, in the form of fruit and muesli bars in addition to drinking water. The final test period consisted of 4 blocks of 4 mins of exercise, in which each of the conditions was presented once. Between these blocks was a rest period of 10 mins so that the device set-up could be changed in time. The current study will report on this final test period only. The first 45 s of the last minute of exercise of each block was analyzed to ensure a steady state in each of the four modes.

### Ergometer

**Figure 2:**
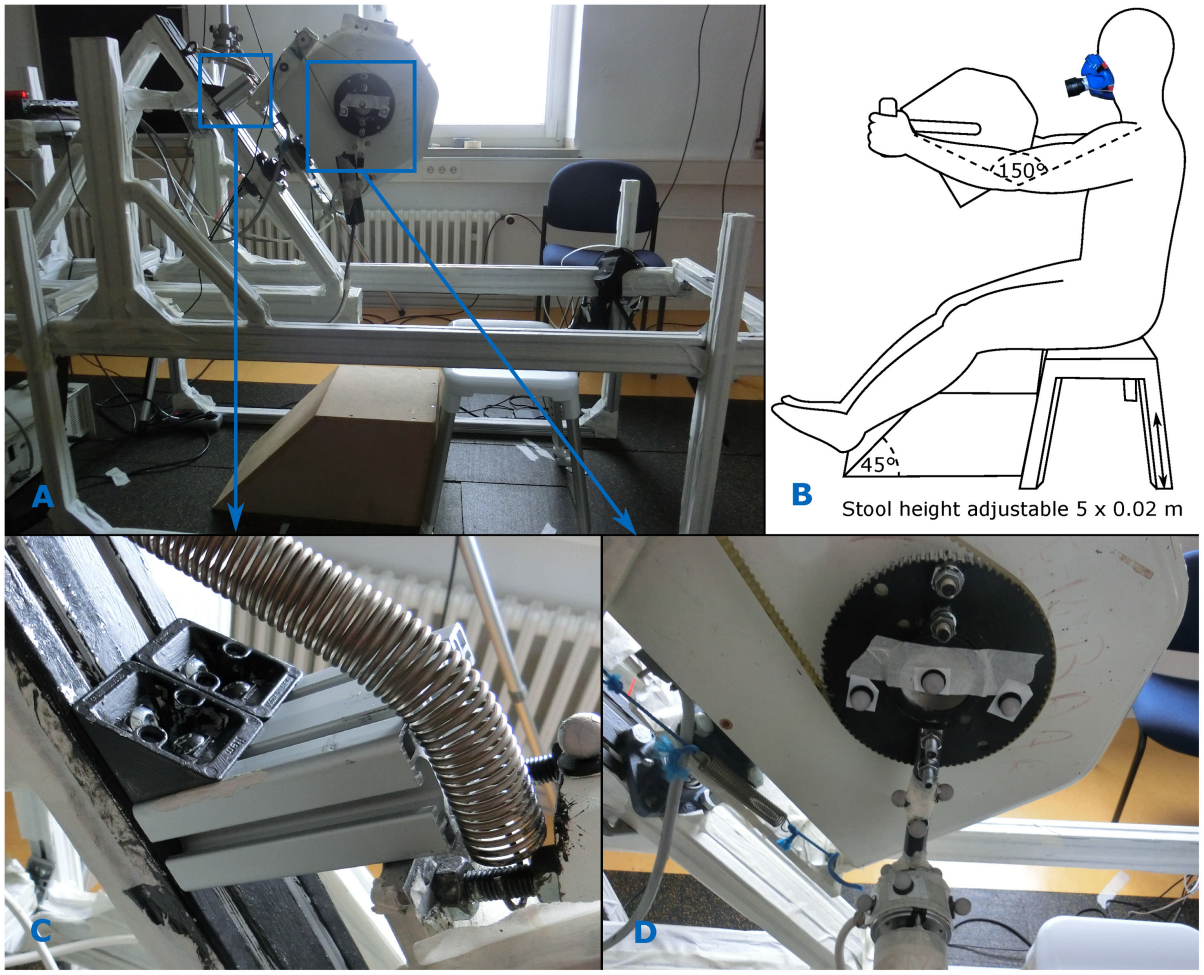
Experimental set-up. **(A)** The instrumented handcycle ergometer. **(B)** The seating position was adjusted so that the shoulder was at or slightly above the crank axis and the elbow was slightly bend when the crank was in the farthest position. **(C)** Placing a block on top of the ergometer prevented the ergometer from rotating. **(D)** The crank mode could be changed by bolting the crank on either side of the blade.

All measurements were done with a custom build stationary instrumented handcycle simulator that was developed at KU Leuven (Verellen et al., 2012a). Instrumented handle bars and cranks were mounted to the crank axis of an electro-magnetically braked ergometer flywheel (Figure 2A). Steering was simulated by using a steering axis mounted with a caster angle of 57° and a damper (spring) between the frame and ergometer. The steer axis could be fixed by adding an extra block between the ergometer and the framework, making it impossible to rotate around the steer axis (Figure 2C). The crank mode could be altered by changing the position of the left crank. The left crank was mounted on an additional blade, which made it possible to create both a synchronous and a synchronous crank mode (Figure 2D). This, however, made that the horizontal distance of the left handle to the center of the crank axis was 0.30 m, while it was 0.28 m for the right handle (same for all conditions). The cranks had a length of 0.17 m. Rather than the sports handcycle as described by Verellen et al. (2012a), an arm-powered add-on handcycle was simulated in the current study by lifting the simulator off the ground with an additional framework (Figure 2A).

The ergometer itself was an electro-magnetically braked system that provided a controlled power output, independent of the crank velocity (Ergometry system 380, Elema Schönanden, Sweden) (Maxwell et al., 1998). The power output could be adjusted from 20 to 500 W in steps of 5 W up to 250 W and in steps of 10 W up to 500 W. In the current experiment, the resistance on the ergometer was set to 35 W, while the instructed crank velocity was 6.3 rad/s (60 rpm). This mean external power output level with a cadence of 60 rpm was assumed to represent daily handcycling on asphalt without curves at a velocity of 3.3 m/s (≈12 km/h) (Hettinga et al., 2013).

### Participant Set-Up

The seating position was adjusted for every participant at the start of the protocol. The stool used in the experiment had no backrest and its height could be adjusted in five steps of 2 cm (Figures 2A,B). It was set at a height so that the shoulder was at or slightly above the height of the crank axis (Hopman et al., 1995; Faupin et al., 2006; Verellen et al., 2008; Krämer et al., 2009). The stool was placed so far back, that the arms were slightly bend (150°) when the hands were on the handles and one of the handles was at the farthest position (Faupin et al., 2006; Goosey-Tolfrey et al., 2008; van Drongelen et al., 2009; Arnet et al., 2014). The initial position was determined with the cosine rule, with the upper and lower arm length as the two known sides, and was adjusted upon comfort after rotating a few full cycles (Figure 2B).

Since the feet of able-bodied participants can be used for stabilization of the trunk (Smith et al., 2008) as well as for propulsion (Smith et al., 2008; Kouwijzer et al., 2018a), the participants' calves rested on a block placed in front of the stool (Figures 2A,B). In that way, more active control and stabilization of the trunk is needed from the upper-body, similar to people with a lower-limb impairment.

Participants were instructed to keep the crank system as straight as possible in a natural manner during exercise, as if they would ride in a straight line. To have an external focus point, a flash light was attached on top of the crank system. The light shone on a white plane in front of the ergometer, and the participants were instructed to keep the light beam centered at the best of their ability. The second instruction was to keep the pace at 60 rpm, which was secured through a metronome. Other than that, no instructions were given, so that a process of natural learning would take place (Vegter et al., 2015; de Klerk et al., 2018; Kraaijenbrink et al., 2020a).

### Physiological Measures

A breath-by-breath respiratory gas analyzer was used to record the pulmonary functions throughout the experiment (Cortex Metax3B, Cortex, Germany; Figure 2B). The system was calibrated with a calibration gas (5% CO_2_, 15% O_2_, BAL. N_2_), as well as a certified 3-L calibration syringe before every participant was measured. Energy expenditure (EE, kcal/h) and energy expenditure per kg (EE, kcal/h/kg) were calculated by the software provided by the manufacturer (Energy Metabolism for CPET in MetaSoft). In addition, oxygen uptake per kg (VO_2_, ml/min/kg), ventilation per kg (VE, L/min/kg), respiratory exchange ratio (RER) and breathing frequency (BF, /min) were exported for further analysis. To ensure a physiological steady-state, the average values of the first 45 s of the last minute of exercise (3.00–3.45) were calculated. The average values per participant were calculated as the weighted arithmetic mean, taking the time between breaths into account. Mechanical efficiency (ME, %) was calculated according to Equation 1. The energy expenditure was multiplied with 1.163 to convert to Watts (W). External power output [PO_propulsion_ (W)] was calculated from the kinetic and kinematic data, as discussed below.


(1) $$ME\;(\%)=\;\frac{PO_{propulsion,\;mean}\;(W)}{EE_{mean}\;(kcal/h)}*100\;\%$$

After each block of exercise, i.e. in the rest periods, the rate of perceived exertion was recorded [RPE, Borg 6-20 (Borg, 1970)].

### Kinetic and Kinematic Measures

Each side of the ergometer was instrumented with a 3D force sensor (FS6, AMTI, USA) and an optical angular encoder (Incremental Shaft Encoders Type RI 58, Hengstler, Germany). The kinetic data were recorded at 100 Hz by custom written data acquisition software (Labview 8.5, National Instruments, USA). Prior to every block of exercise, the force was set to zero, thus removing the off-set of the force measurements.

**Figure 3:**
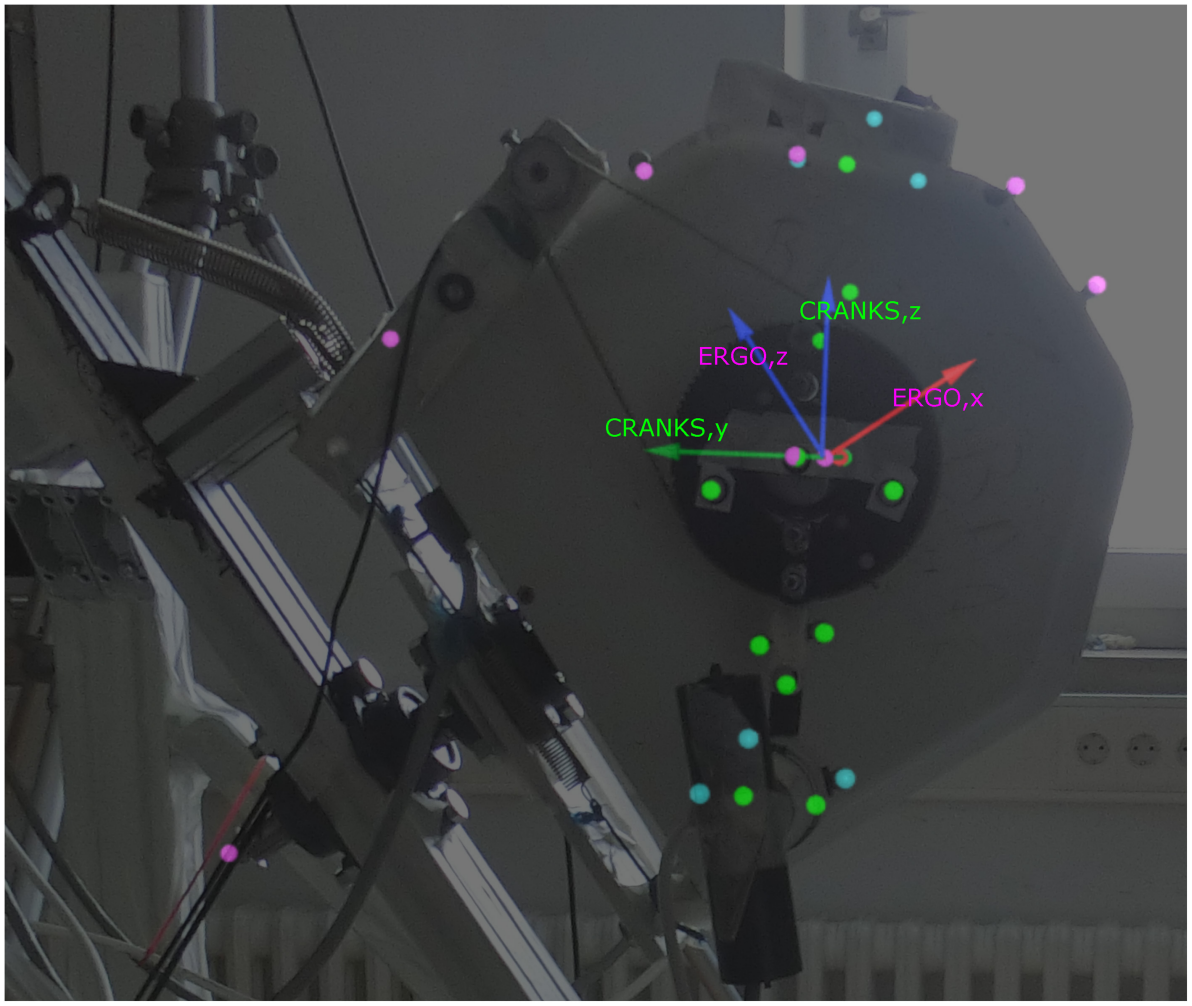
Kinematic markers and the coordinate systems of the rigid bodies as shown in QTM. Lime, rigid body markers of CRANKS; Fuchsia, rigid body markers of ERGO; Aqua, markers on optical encoder.

In order to determine the spatial position of the ergometer and the cranks, motion capture was performed with 8 infrared detecting cameras (Miqus, Qualisys, Sweden) and the according software [Qualisys Track Manager (QTM)]. Infrared reflecting markers were placed on the ergometer, on both cranks and on both optical encoders (Figure 3). 3D kinematic data was collected at 300 Hz. Within the software environment rigid bodies (6DOF) were defined, from which the rotational and translational movements are described. One rigid body (CRANKS) was defined with one of the axes alongside the crank axis. The roll of this rigid body was equal to the rotation of the cranks, i.e., defined the crank angle. The zero was defined when the right crank was vertical (6 o'clock). A second rigid body (ERGO) was defined with one of the axes along the steering axis. The pitch of this rigid body was equal to the steering angle of the system.

### Data Analysis

The kinetic and kinematic data of the first 45 s of the last minute of each of the four modes in the test period were further analyzed in custom written scripts using Matlab (Matlab R2020b, Mathworks, USA). In order to synchronize all kinetic and kinematic signals, the right force sensor was hit with a wooden block that had a marker attached to it, at the start and the end of each measurement. The moment of the hit with the wooden block was gained from visual inspection of both the raw data of this force sensor as well as the kinematic data of the marker using the function “ginput.” The kinetic data was filtered with a second order low-pass recursive Butterworth filter with a cut-off frequency of 10 Hz, as determined by a fast Fourier transform analysis. The kinematic data was down sampled to 100 Hz to fit the kinetic data. Hereafter, the first 45 s of the fourth minute of exercise was selected to match the physiological data. The moment of the hit was used to define these 45 s, so that it also synchronized the kinetic and kinematic data. For both the crank and the steering angle the missing values were filled with the “pchip” method and all end values were extrapolated (“fillmissing”). Subsequently, the data was also filtered with a second order low-pass recursive Butterworth filter with a cut-off frequency of 10 Hz. At this stage, we had the following variables: The force components from the left and right side, the angle of the handlebar from both sides, the crank angle and the steering angle.

The force components were measured on both the left and right side in the local coordinate system of the force transducer. To describe the force components in the cranks' coordinate system, i.e., the tangential (F_Tan_), radial (F_Rad_) and mediolateral force component (F_Lat_), the handlebar angle was used. Subsequently, the radial force component was multiplied by −1 in order to change the positive direction of this force component, to match our previous experiment for better comparison (Kraaijenbrink et al., 2020a). The tangential force component was described as perpendicular to the crank and was positive in the rotating direction of the cranks. The radial force component was directed along the crank and was positive directed toward the crank axis. For both cranks was the mediolateral force component positive when directed horizontally to the left, when the participant was sitting behind the ergometer. Afterwards, the resultant force was calculated and the fraction of effective force (FEF, %) was defined according to Equation 2 (Veeger et al., 1992).


(2) $$FEF\;(\%)=\;\frac{F_{Tan}\;(N)}{F_{resultant}\;(N)}*100\%$$

The torque around the crank axis, was determined from the tangential force component and the length of the crank (Equation 3).


(3) $$\tau_{crank}(Nm)=\;F_{Tan}(N)*length_{crank}\left(m\right)$$

The external power output (PO_propulsion_, W) that was produced around the crank axis during handcycling was determined according to Equation 4 for both sides separately. The angular velocity of the crank (ω_crank_, rad/s) was determined as the first derivative of the crank angle.


(4) $$PO_{propulsion}\left(W\right)=\tau_{crank}(Nm)*\omega_{crank}(rad/s)$$

In order to calculate the torque around the steering axis a similar approach as for the torque around the crank axis was adopted. The torque arm (r_steer_) was defined as the perpendicular distance from the handlebar to the steering axis and was determined throughout the cycle with the use of the kinematic markers as depicted in Figure 3, automatically taking distance to the axis center and the difference between the left and right side into consideration. The torque was determined by the torque arm and the force component that is perpendicular to the steering axis (Equation 5). The force in the local coordinate system of the transducer was transformed with use of the handlebar angle, the crank angle, and the steer angle to gain this force component (F_perp,steer_).


(5) $$\tau_{steer}(Nm)=\;F_{perp,\;steer}\left(N\right)*r_{steer}\;(m)$$

Lastly, the power production around the steer axis (PO_steer_, W) was calculated according to Equation 6. The angular velocity around the steer axis was determined as the first derivative of the steer angle.


(6) $$PO_{steer}\left(W\right)=\tau_{steer}\left(Nm\right)*\omega_{steer}\;(rad/s)$$

**Figure 4:**
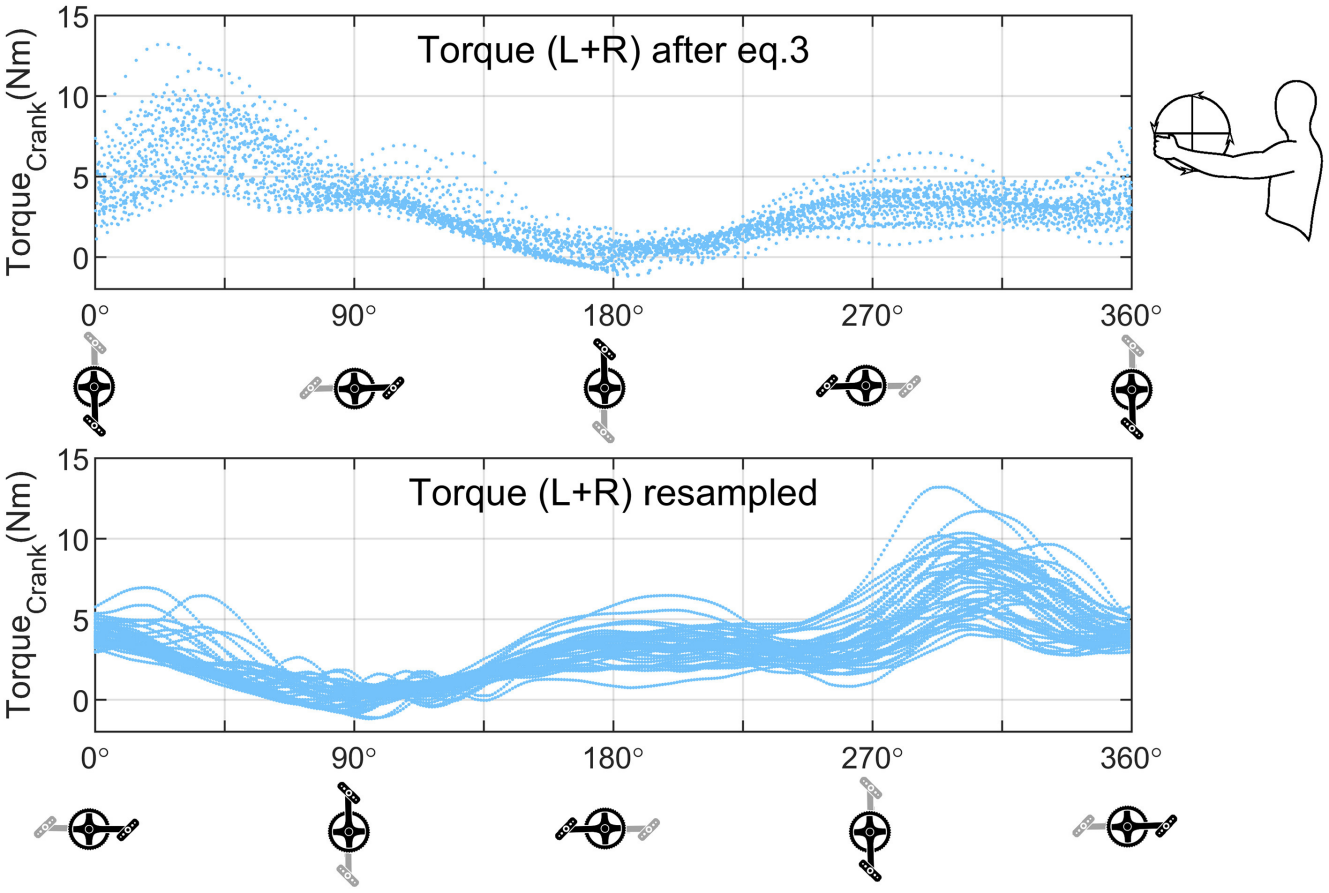
Example resampling of the data. Top graph: The torque values as were calculated in Equation 3 (the sum of the left and right side). Bottom graph: The torque values of the top graph were resampled and shifted over the crank angle to match previous research (Arnet et al., 2012; Kraaijenbrink et al., 2020a). The values for the synchronous steer condition of one participant (p7) are given as an example.

This complete procedure was repeated for both sides, after which both sides were added (over time) to gain the total force, torque or external power output values produced. The data was resampled to one sample per degree (“interp1” with “pchip” method), as shown in Figure 4. At this step, the crank angle was redefined, so that 0° would be defined when the left crank would be horizontal toward the participant. This was done to match the current data to previous research (Arnet et al., 2012; Kraaijenbrink et al., 2020a). The propulsion cycle could hereafter be divided in six phases: the push up (30–90°), push down (90–150°), press down (150–210°), pull down (210–270°), pull up (270–330°) and the lift up phase (330–30°) (Krämer et al., 2009; Arnet et al., 2012; Verellen et al., 2012c; Kraaijenbrink et al., 2020a).

To check whether the instructions given to the participants were effective, the amount of steering and the cadence were also calculated. The amount of steering was determined as the range of the steering angle over the cycle, after which the average over all cycles was calculated. The cadence was determined according to Equation 7.


(7) $$Cadence\;\left(rpm\right)=\frac{\;\omega_{crank}\;(rad/s)}{\left(2*\pi /60\right)}$$

### Statistical Analysis

For one of the participants (P16), the force production was found to be non-realistic after visual inspection of the force production pattern. In one of the conditions the force on the right side was directed perpendicular to what seen on the left side or in the other participant, suggesting that an error was made during the measurement. Therefore, the data of this participant were excluded from all the statistical analyses. The statistical analysis of all the physiological outcomes for each of the four conditions in the test period, as well as the mean propulsion and steering power, the amount of steering and the cadence was performed in SPSS (IBM SPSS Statistics for Windows, Version 27.0, Armonk, NY: IBM Corp). All parameters were checked for normality with Q-Q plots and the Shapiro-Wilk test.

For the non-normally distributed variables, ME (%), RER, RPE, the mean steering power (W), the amount of steering (°) and the cadence (rpm), two separate Wilcoxon Sign Rank Tests were performed. In the first test both steering conditions, i.e., the fixed (Syn and Asyn Fixed; *n* = 30) vs. free steering axis conditions (Syn and Asyn Steer; *n* = 30), were compared independent of the crank mode. In the second test both crank modes, synchronous (Syn Fixed and Syn Steer; *n* = 30) and asynchronous (Asyn Fixed and Asyn Steer; *n* = 30), were compared independent of the steering. The significance level was corrected for multiple tests according to Bonferroni so that α = 0.025.

The mean values of EE (kcal/h/kg), VO_2_ (ml/min/kg), VE (L/min/kg), BF (/min) and the mean propulsion power (W) were normally distributed. For these variables a two-way repeated measures ANOVA with the within subjects' factors “steering axis” and “crank mode” was performed. The significance level was set at α = 0.05.

For the comparison of the fraction of effective force as well as the torque and the angular velocity around the crank and steering axis, the statistical parametric mapping strategy was introduced, allowing the comparison of the values over the entire cycle (Pataky, 2012). As the least number of cycles within the 45 s time-window was 38 for one of the participants in one of the conditions, the first 38 cycles were included for all participants, so that 570 (15 × 38) cycles were included for each condition. The function “anova2rm” from the SPM1D package for Matlab was used to perform the two-way repeated measures ANOVA (spm1d package for Matlab version M.0.4.8; Pataky, 2018). The same independent variables and the significance level were set for this type of ANOVA, respectively “steering axis,” “crank mode” and α = 0.05.

## Results

### Physiological Effects

**Figure 5:**
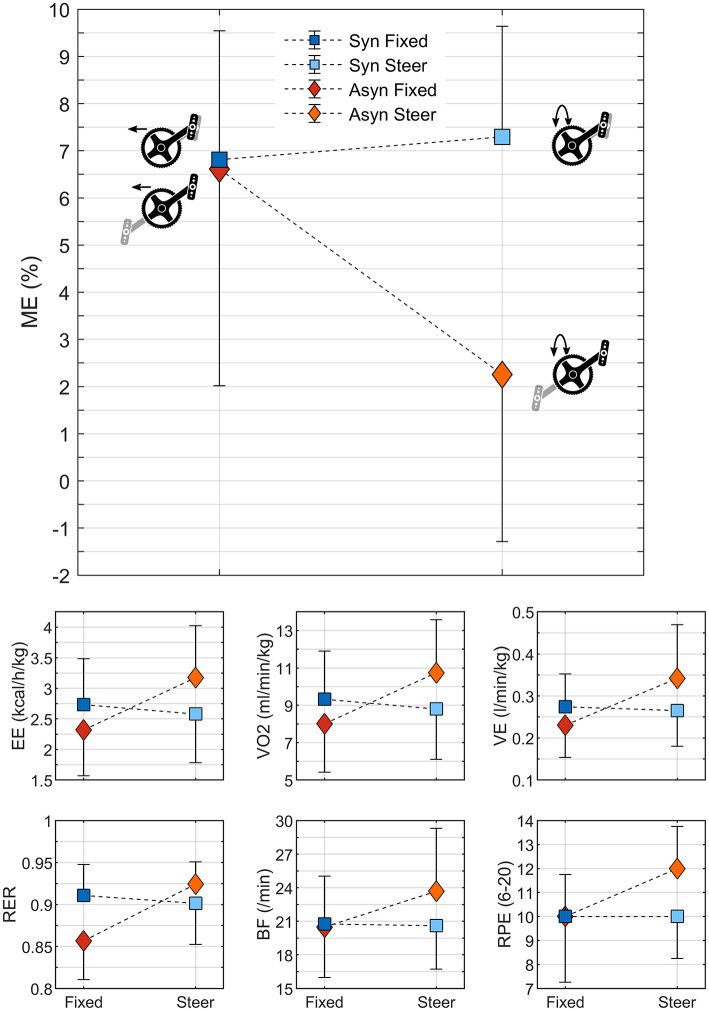
The physiological variables as measured with the Cortex (*n* = 15). For the normally distributed variables, EE, VO_2_, VE, and BF, the mean and standard deviation of the group are presented. For the non-normally distributed variables, ME, RER and RPE, the median and interquartile range are given.

The steering condition had a clear effect on the metabolic response to simulated asynchronous handcycling; this was not seen for synchronous handcycling (Figure 5, Table 1). For all physiological variables, hence ME, EE, VO_2_, VE, RER, BF and RPE, a significant effect of steering was found. Only ME showed a significant effect for the crank mode, due to a difference in propulsion power as discussed in the following sections. Most interestingly, however, is the interaction effect, that could only be determined for the normally distributed variables. For EE, VO_2_, VE and BF an interaction was determined as is evident from Figure 5.

**Table 1:** Statistical outcomes for the physiological variables.

	**Fixed vs. Steer**			**Syn vs. Asyn**			**Interaction**		
	***F*_**(1,14)**_/z**	***P***	$\eta_{\text{p}}^{2}$ **/r**	***F*_**(1,14)**_/z**	***P***	$\eta_{\text{p}}^{2}$ **/r**	***F*_**(1,14)**_**	***P***	$\eta_{\text{p}}^{2}$
ME (%)^a^	−2.99	0.003^*^	−0.55	−3.53	<0.001^*^	−0.64	–	–	–
EE (kcal/h/kg)^b^	47.88	<0.001^†^	0.77	1.14	0.304	0.08	32.63	<0.001^†^	0.70
VO_2_ (ml/min/kg)^b^	42.23	<0.001^†^	0.75	1.28	0.277	0.08	32.03	<0.001^†^	0.70
VE (l/min/kg)^b^	23.81	<0.001^†^	0.63	1.14	0.304	0.08	16.45	0.001^†^	0.54
RER^a^	−3.01	0.003^*^	−0.55	−1.27	0.206	−0.23	–	–	–
BF (/min)^b^	4.86	0.045^†^	0.26	3.52	0.082	0.20	8.16	0.013^†^	0.37
RPE (6–20)^a^	−2.71	0.007^*^	−0.49	−2.21	0.027	−0.40	–	–	–

a *Two Wilcoxon Signed Rank Tests were performed: Fixed vs. Steer (independent of crank mode; n = 30) and Syn vs. Asyn (independent of steering; n = 30)*;

b *One repeated measures ANOVA was performed (n = 15)*;

* *Significant P < 0.025*;

† *Significant P < 0.05; –, No interaction effect could be calculated*.

### Kinetic Effects

**Figure 6:**
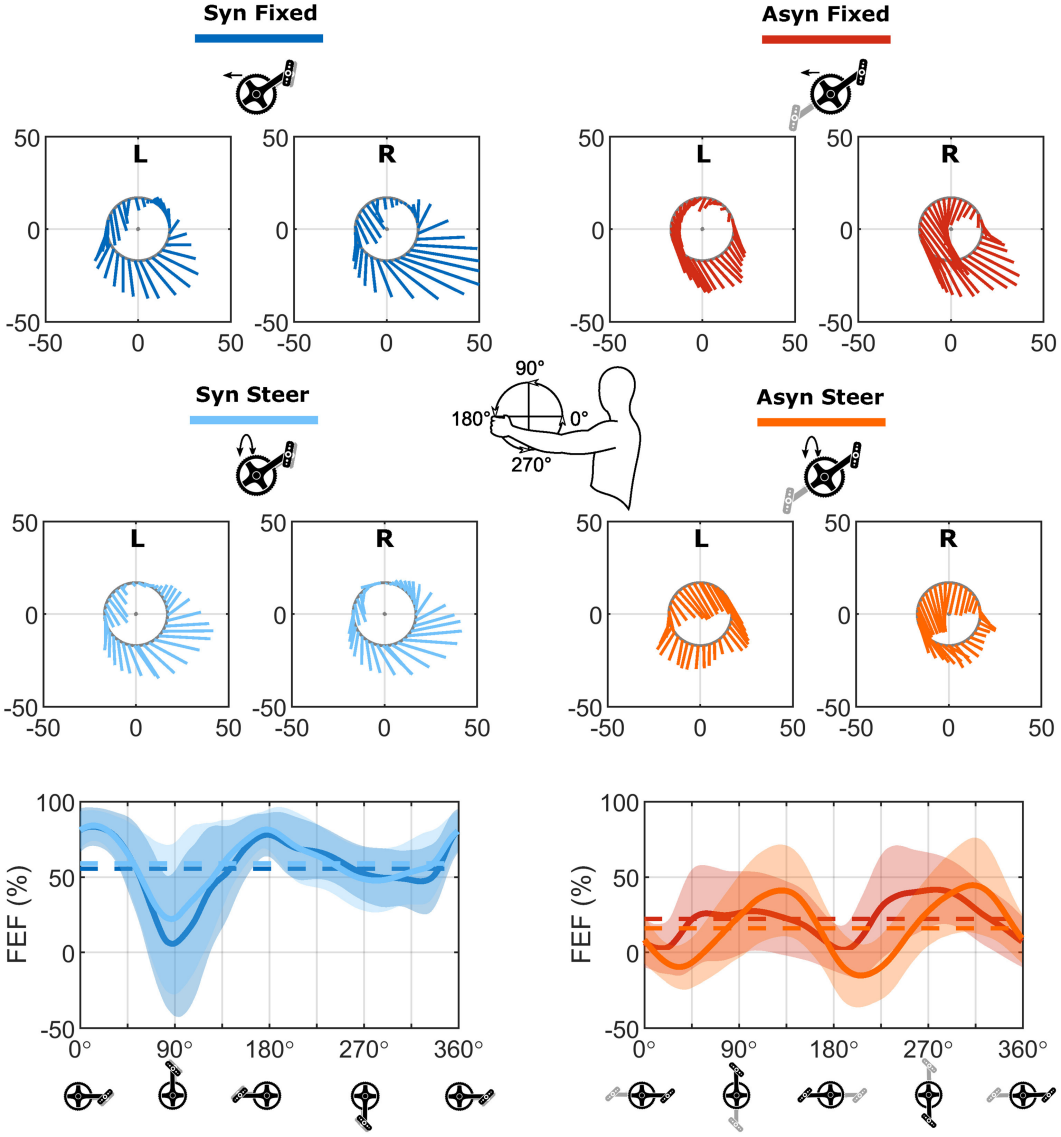
Unilateral force vectors and bilateral fraction of effective force for each of the four conditions. Top eight graphs: Typical example of unilateral force production (shown per 3°) in the local sagittal plane of the crank (resultant F_Tan_ and F_Rad_). One cycle (20th) is shown for one participant (P7) who represents the group average. Both sides are presented separately for each of the four conditions (view left side). Bottom two graphs: The bilateral fraction of effective force calculated as the mean of both sides. The solid line represents the mean cycle of all analyzed cycles of all participants (*n* = 570), the shaded area indicates the standard deviation, whereas the dashed line gives the average values.

**Figure 7:**
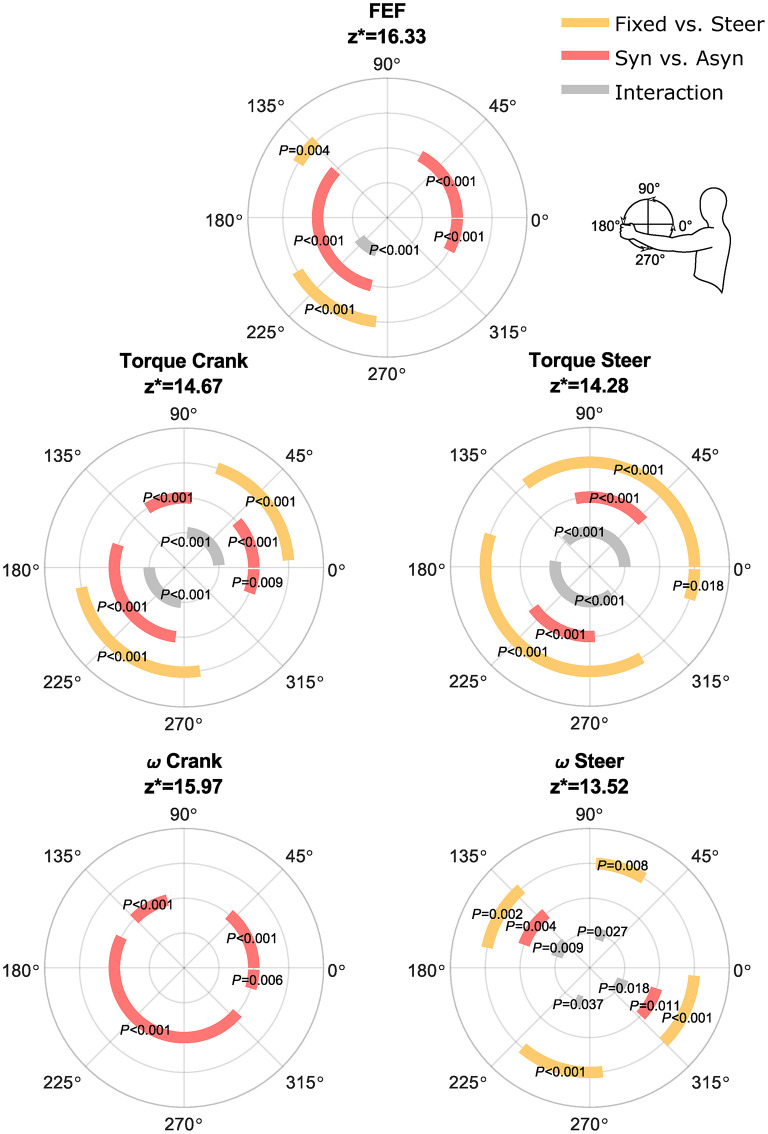
Outcome of statistical analyses performed with the SPM1D method for all cycles and participants (*n* = 570). From outer to inner: steer effect, crank mode effect, interaction effect. z* = critical statistical test value. The degrees of freedom were (1, 14) for all variables. Whenever a line is presented, the conditions were significantly different from each other over that part of the cycle. E.g., the FEF is significantly different (*P* = 0.004) between the fixed and the steering conditions from 133 until 149° (during the push down phase).

In Figure 6, a typical example of the force vectors over the cycle in the sagittal plane is shown for both sides. In all conditions, except for asynchronous handcycling with steering requirements, a clear pull strategy could be recognized. Whether this force production is effective can be determined by the FEF, which is presented in the bottom graphs of Figure 6. The mean values were lower for the asynchronous conditions (Fixed: 22%, Steer: 16%) in comparison to the synchronous conditions (Fixed: 56%, Steer: 59%). The differences in the pattern of FEF between the fixed and steering conditions were largely due to the effects during asynchronous handcycling. During the push down phase the force production in the steering condition was more effective, whereas the opposite was true during the pull-down phase (Fixed vs. Steer; FEF; Figure 7). Synchronous handcycling was more effective than asynchronous handcycling throughout the cycle. Nevertheless, no differences between modes were found in the push up and push down phases, as force production was also less effective for synchronous handcycling in these two phases (Syn vs. Asyn; FEF; Figure 7).

#### Torque and Angular Velocity for Propulsion and Steering

**Figure 8:**
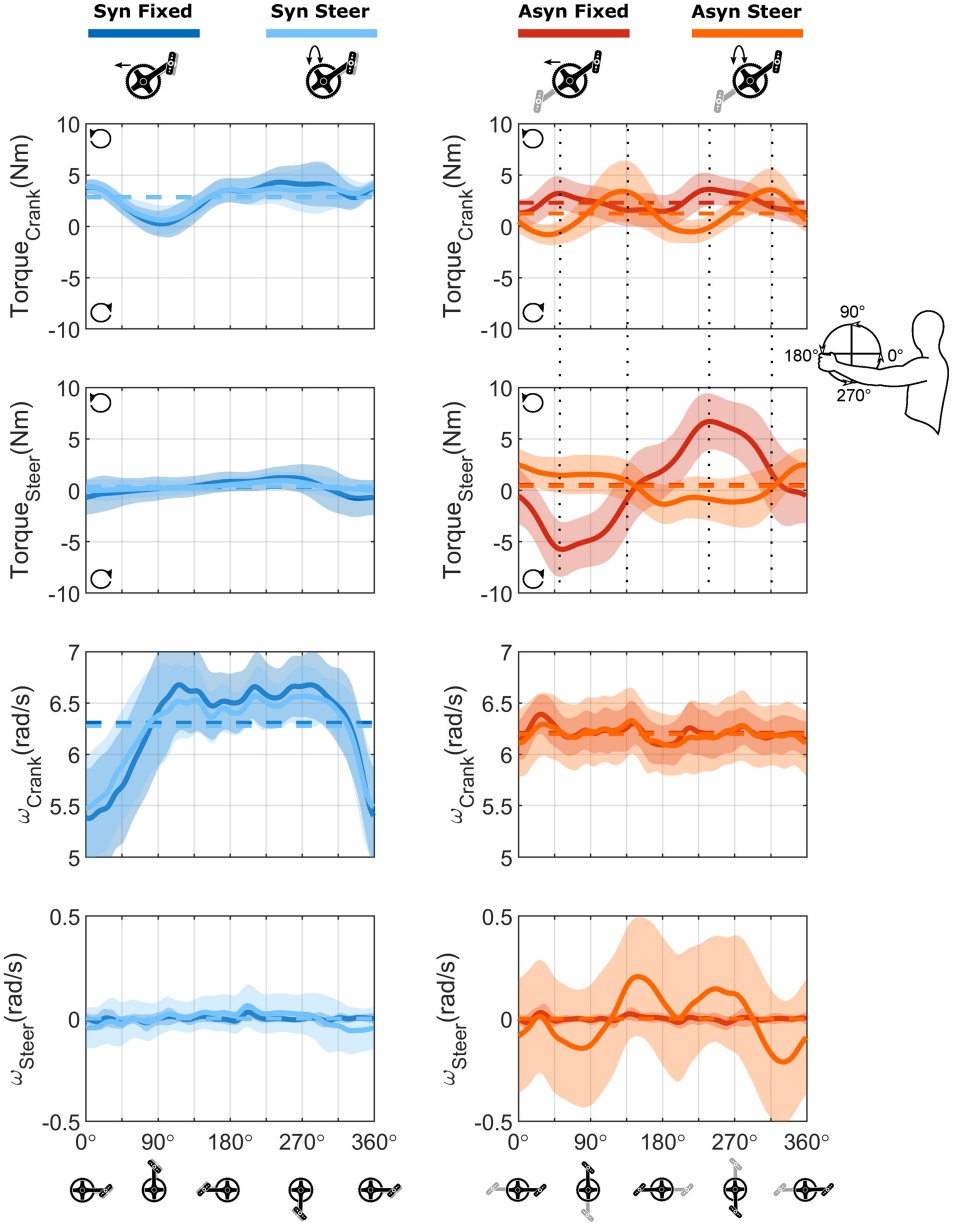
Bilateral torque profiles and velocity profile for each of the four conditions. Top four figures: The bilateral profile of the torque around the crank axis (first row) and the steering axis (second row), calculated as the sum of both sides. Counterclockwise torques were positive, while clockwise directed torques were negative. Bottom four figures: The profile on the velocity around the crank (third row) and the steering axis (fourth row) as determined from the kinematics. The solid line represents the mean cycle of all cycles for all participants (*n* = 570), the shaded area the standard deviation, whereas the average values are represented by the dashed line.

In Figure 8, the torques around the crank and steering axis are shown in the top four graphs. For synchronous and asynchronous handcycling a different handcycle pattern was found (Figure 8—top two graphs), as the torque production around the crank axis was significantly different during the lift up, push down, press down and pull-down phase as defined from the left handlebar (Syn vs. Asyn; Torque Crank; Figure 7).

The torque production during synchronous handcycling was quite similar for both the fixed and steering condition (Figure 8—left). The torque production during asynchronous handcycling, however, was completely different for the fixed and steer conditions (Figure 8—right). The torque around the crank axis was significantly different in the lift-up, push-up, press-down and pull-down phases as defined from the left handlebar (Fixed vs. Steer; Torque Crank; Figure 7). Cause for this difference was the timing of the peak torque amongst others, as a phase shift of about 90° is seen. Whenever the steering axis was fixed, the force produced at the handlebar resulted in a peak torque around the crank axis at the same time as the peak torque around the steering axis. This would result in a large rotation movement, if the steering axis was not fixed. Whenever the peak torque around the crank axis was produced for the steer condition, the steer torque was (close to) zero. Hence the propulsion torque did not result in a rotation movement. The difference between the fixed and steering condition therefore were significant (Fixed vs. Steer; Torque Steer; Figure 7).

**Figure 9:**
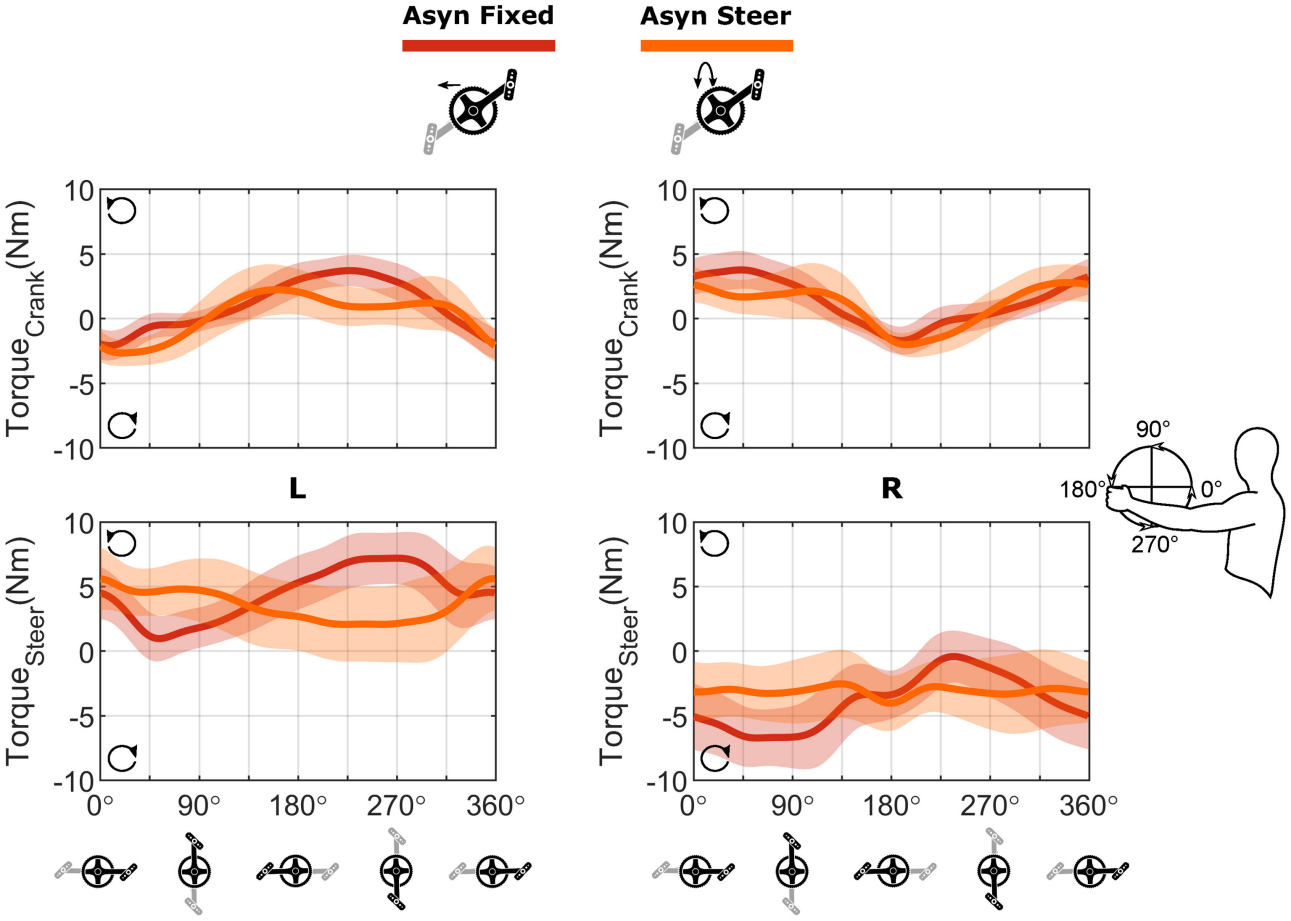
Unilateral torque production (L/R) during asynchronous handcycling with and without the steering requirements. The solid line represents the mean cycle of all cycles for all participants (*n* = 570).

To investigate the torque in more detail, the torque produced at the handlebars is shown separately as well (Figure 9). As is clear from Figure 8, negative propulsion torque was produced while handcycling asynchronously with steering requirements. In Figure 9 it is shown that the positive peak torque was reduced with respect to the fixed condition, while the negative peak remained the same, resulting in a reduced total torque production and a lower overall power production. The steering torque, however, was more constant as a consequence. On the left side one produced a left rotating (counterclockwise) torque, while on the right side a right rotating (clockwise) torque was produced.

For the angular velocity of the cranks, i.e., the result of the produced torque, solely an effect of crank mode was found (ω Crank; Figure 7). An acceleration and deceleration phase were found for the synchronous mode, whereas in the asynchronous mode the cranks' angular velocity was fairly constant (Figure 8). For the angular velocity around the steering axis a mode, steering and interaction effect was found throughout different portions of the cycle (ω Steer; Figure 7). From Figure 8 it is clear that more rotations were present in the asynchronous mode with steering conditions, and this was the only condition that strongly differed from the other three.

### Verifying the Conditions

The first instruction to the participants was to keep the crank system as straight as possible, as if one would ride in a straight line. In the fixed conditions, a small tremble was measured (0.3°), but no real rotations could take place. In the Syn Steer condition the participant only showed small amounts of steering (2.1°), whereas the participants had the most trouble controlling the system in the Asyn Steer condition (6.8°; Table 2). As a consequence, the steering power production was higher in the Asyn Steer condition (−0.09 W) compared to the other three conditions (Table 2). However, only the difference between both crank modes was significant.

**Table 2:** Statistical outcomes for the amount of steering, cadence and power production.

	**Syn fixed**	**Syn steer**	**Asyn fixed**	**Asyn steer**	**Fixed vs. Steer**			**Syn vs. Asyn**			**Interaction**		
					***F*_**(1,14)**_/z**	***P***	$\eta_{\text{p}}^{2}$ **/r**	***F*_**(1,14)**_/z**	***P***	$\eta_{\text{p}}^{2}$ **/r**	***F*_**(1,14)**_**	***P***	$\eta_{\text{p}}^{2}$
Amount of steering (°)^a^	0.3 ± 0.4	2.1 ± 0.5	0.3 ± 0.4	6.8 ± 3.5	−4.78	<0.001^*^	−0.87	−3.88	<0.001^*^	−0.71	–	–	–
Cadence (rpm)^a^	59.6 ± 0.4	59.7 ± 0.3	59.6 ± 0.3	59.5 ± 1.6	−0.67	0.504	−0.12	−1.00	0.318	−0.18	–	–	–
Power steer, mean (W)^a^	0.00 ± 0.01	0.01 ± 0.01	0.00 ± 0.04	−0.09 ± 0.16	−0.67	0.504	−0.12	−2.71	0.007^*^	−0.49	–	–	–
Power propulsion, mean (W)^b^	17.7 ± 1.8	17.4 ± 2.3	14.3 ± 2.2	7.6 ± 5.1	51.37	<0.001^†^	0.79	20.12	0.001^†^	0.59	24.17	<0.001^†^	0.63

a *Two Wilcoxon Signed Rank Tests were performed: Fixed vs. Steer (independent of crank mode; n = 30) and Asyn vs. Syn (independent of steering; n = 30)*;

b *One repeated measures ANOVA was performed (n = 15)*;

* *Significant P < 0.025*;

† *Significant P < 0.05*.

Secondly, the participants were instructed to keep a cadence of 60 rpm with help of a metronome. All 15 participants were able to keep this pace in all four conditions.

Lastly, the ergometer was set at 35 W, however, this value was unexpectedly not produced in any of the conditions. The experimentally measured propulsion power output during synchronous handcycling was around 17 W, and in asynchronous handcycling with a fixed steer axis, the value was within the same range (14.3 W). The mean value in the asynchronous steering condition was the lowest and not within the same range as the other three conditions with only 7 W. Therefore, a significant difference was found between synchronous and asynchronous handcycling, as well as between the fixed and steer conditions.

## Discussion

The current study examined how steering and propelling simultaneously affect the metabolic cost, kinetics and kinematics of submaximal synchronous and asynchronous handcycling. For synchronous handcycling, steering does not affect the handcycle performance whatsoever. In contrast, steering requirements impose participants to change their handcycle technique during asynchronous handcycling, as could be shown with the torque around the crank and steering axis. This again led to an increase in metabolic cost and a reduction in mechanical efficiency. It made asynchronous handcycling with steering the least efficient condition, as hypothesized. As soon as the steering was fixed, there was no need for stabilization and the metabolic cost was the lowest over all four conditions. Yet, as the external propulsion power output was slightly lower than for synchronous handcycling, the mechanical efficiency was at the same level as for the synchronous conditions.

### Physiological Effects

Asynchronous handcycling with steering requirements resulted in the least mechanical efficient condition. This is in line with the handcycling literature, where the experiment were done on a treadmill (van der Woude et al., 2000, 2008; Dallmeijer et al., 2004; Bafghi et al., 2008; Kraaijenbrink et al., 2020a). Moreover, the metabolic response, as determined by EE, VO_2_, VE and RER, showed that asynchronous handcycling without steering requirements resulted in the least amount of energy use, as is in line with the ergometer studies of Hopman et al. (1995) and Goosey-Tolfrey and Sindall (2007). The biomechanics of handcycling, by means of torque patterns and the resulting angular velocity can be used to explain the physiological phenomena found.

### Kinetic Effects

#### Torque and Angular Velocity for Propulsion and Steering

The participants were capable to compensate for steering movements, by changing their cycling technique, however with an increased metabolic cost. The peak torque around the crank axis showed a phase shift of 90°. With this shift in timing, the production of peak propulsion torque resulted in (almost) no steering torque, preventing a rotation of the ergometer. As a consequence, asynchronous handcycling with steering was metabolically less efficient and less effective in terms of FEF, as was previously shown in the literature (van der Woude et al., 2000, 2008; Dallmeijer et al., 2004; Bafghi et al., 2008; Kraaijenbrink et al., 2020a). In the fixed condition, the metabolic costs were lower, yet the cycling technique used in this condition creates great torques around the steering axis. Would this axis not be fixed by the block on top of the ergometer, these torques would result in lots of rotation, making it difficult to propel. To prevent the large rotation, the steering torque needs to be reduced in the condition when the axis is not fixed.

### Verifying the Conditions

Interestingly, the compensation strategy has the consequence that even negative propulsion torque and with-it negative propulsion power production is seen in the Asyn Steer condition (Figure 8). As this is the only condition with this negative power, the total power production is lower for this condition compared to the other three (Table 2). This is in line with the findings of Bafghi et al. (2008) as they showed that the resultant force in the sagittal plane was lower for asynchronous compared to synchronous handcycling over different speeds when cycling on a motorized treadmill. In a previous experiment we also found a lower external power output for asynchronous handcycling compared to synchronous handcycling (Kraaijenbrink et al., 2020a). With practice the external power output increased, but did not reach the value found for synchronous handcycling. In that experiment the participants rode on a motorized level treadmill at equal speed. The differences in external power output were believed to be caused by the movement of the handcycle on the treadmill, but this cannot be the reason based on the current results.

The current experiment as well as our previous experiment indicate that power production works quite differently in synchronous compared to asynchronous handcycling. During synchronous handcycling, the left- and right-hand produce propulsion torque at the same moment in the cycle and no negative torque is being produced within the entire cycle. Contrarily, when the left hand produces positive propulsion torque, hence, propels the cranks, the right side shows a breaking torque during asynchronous handcycling. From the angular velocity (ω_crank_) and the cadence, however, it is clear that the participants have never made a breaking movement. The participants hold the right handlebar in a stable position, while the left hand is pulling for propulsion. Both cranks rotate forward because of this pulling, thus creating a negative propulsion torque on the right side which is held (Figure 9), and vice versa. When comparing the current asynchronous handcycling results to bicycling, in which the cranks are also mounted asynchronously, a similar propulsion profile is seen (Rossato et al., 2008; Bini et al., 2011). In bicycling, two peaks are seen in the overall torque production. At the level of the pedal, it was shown that whenever the left pedal is producing positive torque, the torque in the right pedal is negative and vice versa (Bini et al., 2011). In both the bicycling study and our results for the fixed condition, we see that the positive torque on the one side is higher than the negative torque on the other side (Figure 9—top graphs).

When steering comes into play in the asynchronous mode, some of the propulsion torque and with-it power might be lost due to the steering movements. The steering movements are indeed higher in the Asyn Steer condition (Table 2), whilst the positive peak in propulsion torque reduces and the negative propulsion torque is more prominent (Figure 9—top graphs). The higher steering “range of motion” results in a need for a higher steering power production to keep the ergometer straight. As can been seen in the bottom graphs of Figure 9, one produces left rotation torque on the left side, while producing right rotating torque on the right. Key is to reduce the steering torque peaks and keep the left and right side in balance. In the synchronous conditions a low range of motion is found. In addition, whenever the propulsion torque at the left handlebar is causing a counterclockwise steering torque, the propulsion on the right side causes a clockwise steering torque at exactly the same time. The ergometer is therefore always in balance. Yet, this does not concern the asynchronous mode. Whenever one pulls too hard on one side in this mode, the steering torques will be out of balance and a rotation of the ergometer will be the result, making it even harder to propel. To reduce the peak steering torque, the peak propulsion torque was reduced, with a loss of propulsion power as a result. Interestingly, this has not led to a reduction in cadence and participants were able to keep the instructed pace.

### Limitations

**Figure 10:**
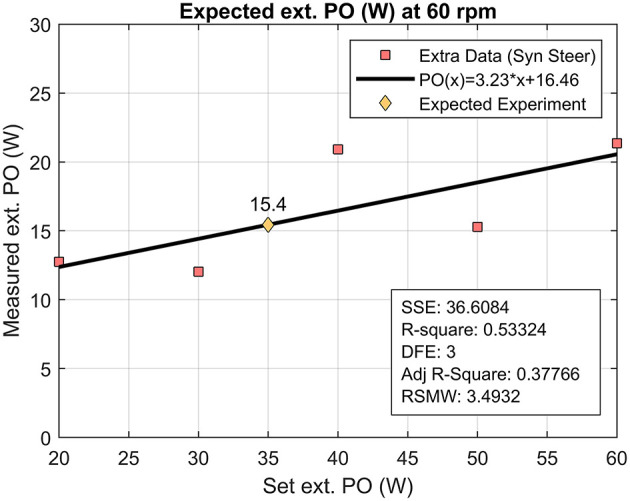
Experimental determination of the external power output of the handcycle ergometer when the device is set to 35 W. The experiment was done while handcycling with 60 rpm in the synchronous mode with steering requirements.

Even though the resistance was set to 35 W in all four conditions, the mean external power output (PO_propulsion_) as determined from the force sensors does not reach this value in any of them. The ergometer used in this experiment is the Ergometry system 380 of Elema Schönanden, Sweden. The device itself was originally a bicycle ergometer that was fit into a frame for handcycle research. During the build of the simulated handcycle ergometer, the ergometer was calibrated (Verellen et al., 2012a). In the previous experiments with the same device, the power was set to 90% of the participants' peak power output (Verellen et al., 2012a, c) or to 130 W (Verellen et al., 2012b). In the current experiment, the lower end of the spectrum was used, as the power on the ergometer can be set from 20 up to 500 W. For bicycling, these extremely low values are not used often. It might be possible that the ergometer is not as accurate for these low values. In a bicycling study, a stationary cycle ergometer was equipped with both the SRM power meter and strain gauge instrumented pedals for the comparison of these data acquisition methods. In comparison to the ergometer, the instrumented pedals underestimated the power output, as only 80% of the total power output was found at 100 W and 86–88% at 150 up to 300 W (Bini et al., 2011). It seems that at a lower total external power output the inaccuracy of the ergometer motor is larger than at higher power outputs. Before the current experiment, the ergometer was not recalibrated. As we did find these values to be lower than was set, additional tests were performed, while the new “expected” power output was fitted (Figure 10). On hindsight, the external power output is believed to be around 15.4 W (prediction bounds: 3.1–27.7 W), although this is a very rough estimate due to the limited data available. The outcomes of the current study are not affected by the absolute value of the external power output, as the ergometer was set at the same value for all exercise blocks. On the other hand, all the measurement equipment, i.e., both force sensors and the optical encoders, were independent of the ergometer and calibrated before the experiment. Therefore, we are confident that the measured forces and with that the calculated external power output is correct.

With the exception of the asynchronous steering condition, the external power output level was around 14–17 W. These low values have previously been found in treadmill-based studies, where the belt speed was set at 1.11–1.94 m/s (Bafghi et al., 2008; van der Woude et al., 2008; Arnet et al., 2013; Kraaijenbrink et al., 2020a). In addition, it was previously shown that an average value of 11.8 W was associated with a velocity of 1.7 m/s for outdoor handcycling with a cadence of 60 rpm (Hettinga et al., 2013). These low external power output values are only expected for daily handcycling, e.g., for commuting. The focus of the current study was therefore on mechanical efficiency and effectiveness. For recreative or athlete sports, in which high speeds are more important and higher external power output levels are reached, the results may not uphold. Future studies should look at the (dis)advantages of both synchronous and asynchronous handcycling at high level submaximal efforts as well as during accelerations, when the difference between winning or losing is made.

As the current experiment was conducted with able-bodied men, the population of handcycle users may not be represented. The current experiment asks for novices, as experience in one of the conditions may affect the performance. It is difficult to ask individuals who are in the process of early rehabilitation to volunteer in an experiment, as they already go through a stressful time (Leving et al., 2019). In order to simulate novice handcycle users however, a block was placed in front of the stool to prevent the participant from pushing off the ground with their feet. In addition, the stool had no backrest making it harder to control, as the backrest could not be used to stabilize the trunk or to push off against. The downside from this is that the participants can change interface or adjust the flexion extension in the trunk between conditions. Therefore, the stool was set at such a height that the crank axis was at shoulder level. As such, the participants would need to employ an arm powered strategy, and will not be able to use their trunk for propulsion, however this cannot be fully canceled out. The knowledge gained from this study, i.e., that steering requirements have an effect on asynchronous, but not on synchronous handcycling, is nevertheless expected to hold for daily handcycle users.

## Conclusion

For daily submaximal handcycling, the synchronous mode is the preferred configuration, as the asynchronous mode will enforce a different handcycle technique due to the steering requirements, followed by a reduction of the propulsion power and an increase in metabolic costs. Researchers, practitioners or handcyclists, who use an arm crank ergometer for practice or exercise testing are urged to use a synchronous crank set-up, as this is closest to the real-life straightforward handcycling.

## Data Availability Statement

The datasets presented in this study can be found in online repositories. The names of the repository/repositories and accession numbers can be found below: DataverseNL repository at: https://doi.org/10.34894/LHGIF1.

## Ethics Statement

The studies involving human participants were reviewed and approved by Local Ethics Committee of the Faculty of Psychology and Sport Science at the University of Muenster. The patients/participants provided their written informed consent to participate in this study. Written informed consent was obtained from the individuals for the publication of any potentially identifiable images or data included in this article.

## Author Contributions

CK, RV, YV, LW, and HW designed the study protocol. CK, NO, and LJ prepared the experimental set-up. CK and NO collected the data. The data analysis was performed by CK, NO, LJ, and HW. Data interpretation by CK, RV, LW, and HW. The first draft was written by CK. RV, LW, and YV contributed in revising the manuscript. All authors read and approved the final manuscript.

## Conflict of Interest

The authors declare that the research was conducted in the absence of any commercial or financial relationships that could be construed as a potential conflict of interest.

## Publisher's Note

All claims expressed in this article are solely those of the authors and do not necessarily represent those of their affiliated organizations, or those of the publisher, the editors and the reviewers. Any product that may be evaluated in this article, or claim that may be made by its manufacturer, is not guaranteed or endorsed by the publisher.

## References

[B1] Abel T. Vega S. R. Bleicher I. Platen P. (2003). Handbiking: physiological responses to synchronous and asynchronous crank montage. Eur. J. Sport Sci. 3, 1–9. 10.1080/17461390300073401

[B2] Arnet U. van Drongelen S. Schlussel M. Lay V. van der Woude L. H. Veeger H. E. (2014). The effect of crank position and backrest inclination on shoulder load and mechanical efficiency during handcycling. Scand. J. Med. Sci. Sports. 24, 386–394. 10.1111/j.1600-0838.2012.01524.x 22989023

[B3] Arnet U. van Drongelen S. Veeger D. H. van der Woude L. H. V. (2013). Force application during handcycling and handrim wheelchair propulsion: an initial comparison. J. Appl. Biomech. 29, 687–695. 10.1123/jab.29.6.687 23343659

[B4] Arnet U. van Drongelen S. Veeger D. H. E. J. van der Woude L. H. (2012). Are the force characteristics of synchronous handcycling affected by speed and the method to impose power? Med. Eng. Phys. 34, 78–84. 10.1016/j.medengphy.2011.07.001 21798789

[B5] Bafghi H. A. de Haan A. Horstman A. Van Der Woude L. (2008). Biophysical aspects of submaximal hand cycling. Int. J. Sports Med. 29, 630–638. 10.1055/s-2007-989416 18213544

[B6] Bini R. R. Hume P. A. Cerviri A. (2011). A comparison of cycling SRM crank and strain gauge instrumented pedal measures of peak torque, crank angle at peak torque and power output. Proc. Eng. 13, 56–61. 10.1016/j.proeng.2011.05.051

[B7] Borg G. (1970). Perceived exertion as an indicator of somatic stress. Scand. J. Rehabil. Med. 2, 92–98. 5523831

[B8] Bresnahan J. J. Farkas G. J. Clasey J. L. Yates J. W. Gater D. R. (2019). Arm crank ergometry improves cardiovascular disease risk factors and community mobility independent of body composition in high motor complete spinal cord injury. J. Spinal Cord Med. 42, 272–280. 10.1080/10790268.2017.1412562 29334345 PMC6522950

[B9] Brizuela G. Sinz S. Aranda R. Martínez-Navarro I. (2020). The effect of arm-crank exercise training on power output, spirometric and cardiac function and level of autonomy in persons with tetraplegia. Eur. J. Sport Sci. 20, 926–934. 10.1080/17461391.2019.1674927 31566476

[B10] Cummins T. D. Gladden L. B. (1983). Responses to submaximal and maximal arm cycling above, at, and below heart level. Med. Sci. Sports Exerc. 15, 295–298. 10.1249/00005768-198315040-00008 6621319

[B11] Dallmeijer A. J. Ottjes L. de Waardt E. Van der Woude L. H. V. (2004). A physiological comparison of synchronous and asynchronous hand cycling. Int J Sport. Med. 25, 622–626. 10.1055/s-2004-817879 15532007

[B12] de Klerk R. Lutjeboer T. Vegter R. J. K. van der Woude L. H. V. (2018). Practice-based skill acquisition of pushrim-activated power-assisted wheelchair propulsion versus regular handrim propulsion in novices. J. Neuroeng. Rehabil. 15:56. 10.1186/s12984-018-0397-4 29940986 PMC6020202

[B13] Faupin A. Gorce P. Campillo P. Thevenon A. Remy-Neris O. (2006). Kinematic analysis of handbike propulsion in various gear ratios: implications for joint pain. Clin. Biomech. 21, 560–566. 10.1016/j.clinbiomech.2006.01.001 16510220

[B14] Faupin A. Gorce P. Meyer C. (2011). Effects of type and mode of propulsion on hand-cycling biomechanics in nondisabled subjects. J. Rehabil. Res. Dev. 48, 1049–1060. 10.1682/JRRD.2010.19.0199 22234710

[B15] Fink K. (1976). Vergleichende leistungsphysiologische Untersuchungen zur Frage des Hebel-oder Kurbelantriebs von handbetriebenen Strassenselbstfahrern (Doctoral dissertation).

[B16] Goosey-Tolfrey V. L. Alfano H. Fowler N. (2008). The influence of crank length and cadence on mechanical efficiency in hand cycling. Eur. J. Appl. Physiol. 102, 189–194. 10.1007/s00421-007-0576-7 17909841

[B17] Goosey-Tolfrey V. L. Sindall P. (2007). The effects of arm crank strategy on physiological responses and mechanical efficiency during submaximal exercise. J. Sports Sci. 25, 453–460. 10.1080/02640410600702883 17365532

[B18] Hettinga F. J. de Groot S. van Dijk F. Kerkhof F. Woldring F. van der Woude L. (2013). Physical strain of handcycling: an evaluation using training guidelines for a healthy lifestyle as defined by the American College of Sports Medicine. J. Spinal Cord Med. 36, 376–382. 10.1179/2045772313Y.0000000127 23820153 PMC3758534

[B19] Hopman M. T. E. van Teeffelen W. M. Brouwer J. Houtman S. Binkhorst R. A. (1995). Physiological responses to asynchronous and synchronous arm-cranking exercise. Eur. J. Appl. Physiol. Occup. Physiol. 72, 111–114. 10.1007/BF00964124 8789580

[B20] Kouwijzer I. Nooijen C. Breukelen K. Janssen T. Groot S. (2018a). Effects of push-off ability and handcycle type on handcycling performance in able-bodied participants. J. Rehabil. Med. 50, 563–568. 10.2340/16501977-2343 29756632

[B21] Kouwijzer I. Valent L. Osterthun R. van der Woude L. de Groot S. on behalf of the H. Group (2018b). Peak power output in handcycling of individuals with a chronic spinal cord injury: predictive modeling, validation and reference values. Disabil. Rehabil. 42, 400–409. 10.1080/09638288.2018.1501097 30507314

[B22] Kraaijenbrink C. Vegter R. de Groot S. Arnet U. Valent L. Verellen J.. (2020b). Biophysical aspects of handcycling performance in rehabilitation, daily life and recreational sports; a narrative review. Disabil. Rehabil. 2020, 1–15. 10.1080/09638288.2020.1815872 32905740

[B23] Kraaijenbrink C. Vegter R. J. K. Hensen A. H. R. Wagner H. van der Woude L. H. V. (2020a). Biomechanical and physiological differences between synchronous and asynchronous low intensity handcycling during practice-based learning in able-bodied men. J. Neuroeng. Rehabil. 17, 1–13. 10.1186/s12984-020-00664-8 32093732 PMC7038515

[B24] Krämer C. Schneider G. Böhm H. Klöpfer-Krämer I. Senner V. (2009). Effect of different handgrip angles on work distribution during hand cycling at submaximal power levels. Ergonomics 52, 1276–1286. 10.1080/00140130902971916 19626501

[B25] Krops L. Albada T. Woude L. Hijmans J. Dekker R. (2017). Anaerobic exercise testing in rehabilitation: a systematic review of available tests and protocols. J. Rehabil. Med. 49, 289–303. 10.2340/16501977-2213 28350415

[B26] Leving M. T. de Groot S. Woldring F. A. B. Tepper M. Vegter R. J. K. van der Woude L. H. V. (2019). Motor learning outcomes of handrim wheelchair propulsion during active spinal cord injury rehabilitation in comparison with experienced wheelchair users. Disabil. Rehabil. 43, 1429–1442. 10.1080/09638288.2019.1668484 31656102

[B27] Maxwell B. F. Withers R. T. Ilsley A. H. Wakim M. J. Woods G. F. Day L. (1998). Dynamic calibration of mechanically, air- and electromagnetically braked cycle ergometers. Eur. J. Appl. Physiol. Occup. Physiol. 78, 346–352. 10.1007/s004210050430 9754975

[B28] Mossberg K. Willman C. Topor M. Crook H. Patak S. (1999). Comparison of asynchronous versus synchronous arm crank ergometry. Spinal Cord. 37, 569–574. 10.1038/sj.sc.3100875 10455533

[B29] Müller E. A. Müller A. (1949). Die günstigste Grösse und Anordnung von Handrädern. Arbeitsphysiologie 14, 27–44. 10.1007/BF00935570

[B30] Pataky T. (2018). Introduction—spm1d 0.4 Documentation. Available online at: http://www.spm1d.org/ (accessed January 30, 2019).

[B31] Pataky T. C. (2012). One-dimensional statistical parametric mapping in Python. Comput. Methods Biomech. Biomed. Eng. 15, 295–301. 10.1080/10255842.2010.527837 21756121

[B32] Rossato M. Bini R. R. Carpes F. P. Diefenthaeler F. Moro A. R. (2008). Cadence and workload effects on pedaling technique of well-trained cyclists. Int. J. Sports Med. 29, 746–752. 10.1055/s-2008-1038375 18302076

[B33] Smith P. M. Chapman M. L. Hazlehurst K. E. Goss-Sampson M. A. (2008). The influence of crank configuration on muscle activity and torque production during arm crank ergometry. J. Electromyogr. Kinesiol. 18, 598–605. 10.1016/j.jelekin.2006.12.006 17337211

[B34] Thomas S. Reading J. Shephard R. J. (1992). Revision of the physical activity readiness questionnaire (PAR-Q). Can. J. Sport Sci. 17, 338–345. 1330274

[B35] van der Woude L. Bosmans I. Bervoets B. Veeger D. H. (2000). Handcycling: different modes and gear ratios. J. Med. Eng. Technol. 24, 242–249. 10.1080/030919000300037168 11315650

[B36] van der Woude L. Horstman A. Faas P. Mechielsen S. Bafghi H. A. de Koning J. J. (2008). Power output and metabolic cost of synchronous and asynchronous submaximal and peak level hand cycling on a motor driven treadmill in able-bodied male subjects. Med. Eng. Phys. 30, 574–580. 10.1016/j.medengphy.2007.06.006 17709272

[B37] van der Woude L. H. V. de Groot G. Hollander A. P. van Ingen Schenau G. J. Rozendal R. H. (1986). Wheelchair ergonomics and physiological testing of prototypes. Ergonomics 29, 1561–1573. 10.1080/00140138608967269 3102225

[B38] van Drongelen S. Maas J. C. Scheel-Sailer A. Van Der Woude L. H. V. (2009). Submaximal arm crank ergometry: Effects of crank axis positioning on mechanical efficiency, physiological strain and perceived discomfort. J. Med. Eng. Technol. 33, 151–157. 10.1080/13561820802565676 19205993

[B39] Veeger D. H. van der Woude L. H. V. Rozendal R. H. (1992). Effect of handrim velocity on mechanical efficiency in wheelchair propulsion. Med. Sci. Sports Exerc. 24, 100–107. 10.1249/00005768-199201000-00017 1548983

[B40] Vegter R. J. K. de Groot S. Lamoth C. J. Veeger D. H. E. J. van der Woude L. H. V. (2014). Initial skill acquisition of handrim wheelchair propulsion: a new perspective. IEEE Trans. Neural Syst. Rehabil. Eng. 22, 104–113. 10.1109/TNSRE.2013.2280301 24122567

[B41] Vegter R. J. K. Hartog J. de Groot S. Lamoth C. J. Bekker M. J. van der Scheer J. W.. (2015). Early motor learning changes in upper-limb dynamics and shoulder complex loading during handrim wheelchair propulsion. J. Neuroeng. Rehabil. 12, 1–14. 10.1186/s12984-015-0017-5 25889389 PMC4367846

[B42] Verellen J. Janssens L. Meyer C. Vanlandewijck Y. (2012a). Development and application of a handbike ergometer to measure the 3D force generation pattern during arm crank propulsion in realistic handcycling conditions. Sport. Technol. 5, 65–73. 10.1080/19346182.2012.754894

[B43] Verellen J. Meyer C. Janssens L. Vanlandewijck Y. (2012b). Peak and submaximal steady-state metabolic and cardiorespiratory responses during arm-powered and arm-trunk-powered handbike ergometry in able-bodied participants. Eur. J. Appl. Physiol. 112, 983–989. 10.1007/s00421-011-2051-8 21717120

[B44] Verellen J. Meyer C. Janssens L. Vanlandewijck Y. (2012c). The impact of spinal cord injury lesion level on force generation effectiveness during handcycling, in Analysis of Performance Determinants in Handcycling (Leuven), 79–94.

[B45] Verellen J. Meyer C. Reynders S. Van Biesen D. Vanlandewijck Y. (2008). Consistency of within-cycle torque distribution pattern in hand cycling. J. Rehabil. Res. Dev. 45, 1295–1302. 10.1682/JRRD.2007.12.0205 19319754

[B46] Williams A. M. M. M. Chisholm A. E. Lynn A. Malik R. N. Eginyan G. Lam T. (2019). Arm crank ergometer “spin” training improves seated balance and aerobic capacity in people with spinal cord injury. Scand. J. Med. Sci. Sports. 30, 361–369. 10.1111/sms.13580 31621945

